# Chloroplast Genome Differences between Asian and American *Equisetum arvense* (Equisetaceae) and the Origin of the Hypervariable *trnY-trnE* Intergenic Spacer

**DOI:** 10.1371/journal.pone.0103898

**Published:** 2014-08-26

**Authors:** Hyoung Tae Kim, Ki-Joong Kim

**Affiliations:** Division of Life Sciences, School of Life Sciences and Biotechnology, Korea University, Seoul, Korea; Leibniz Institute of Plant Biochemistry, Germany

## Abstract

Comparative analyses of complete chloroplast (cp) DNA sequences within a species may provide clues to understand the population dynamics and colonization histories of plant species. *Equisetum arvense* (Equisetaceae) is a widely distributed fern species in northeastern Asia, Europe, and North America. The complete cp DNA sequences from Asian and American *E. arvense* individuals were compared in this study. The Asian *E. arvense* cp genome was 583 bp shorter than that of the American *E. arvense*. In total, 159 indels were observed between two individuals, most of which were concentrated on the hypervariable *trnY-trnE* intergenic spacer (IGS) in the large single-copy (LSC) region of the cp genome. This IGS region held a series of 19 bp repeating units. The numbers of the 19 bp repeat unit were responsible for 78% of the total length difference between the two cp genomes. Furthermore, only other closely related species of *Equisetum* also show the hypervariable nature of the *trnY-trnE* IGS. By contrast, only a single indel was observed in the gene coding regions: the *ycf1* gene showed 24 bp differences between the two continental individuals due to a single tandem-repeat indel. A total of 165 single-nucleotide polymorphisms (SNPs) were recorded between the two cp genomes. Of these, 52 SNPs (31.5%) were distributed in coding regions, 13 SNPs (7.9%) were in introns, and 100 SNPs (60.6%) were in intergenic spacers (IGS). The overall difference between the Asian and American *E. arvense* cp genomes was 0.12%. Despite the relatively high genetic diversity between Asian and American *E. arvense*, the two populations are recognized as a single species based on their high morphological similarity. This indicated that the two regional populations have been in morphological stasis.

## Introduction

Approximately 480 complete chloroplast (cp) genome sequences are currently publicly available (http://www.ncbi.nlm.nih.gov/genome), the majority of which are derived from economically important crop plants. Comparative analysis of chloroplast sequences indicate that genome structure, gene content, and gene order are largely stable in land plant lineages [1]. However, highly rearranged cp genome structures are observed in some land plant lineages, and can be used as molecular markers to elucidate the ancient divergence of specific groups [2]–[4]. Because of the generally conservative nature of cp genome structure, cp genome data are used most often to address phylogenetic and evolutionary questions at or above the species level. Nevertheless, base substitutions and small indels are seen frequently in cp genomes, even between closely related taxa. [3], [5], [6].

Cp genome comparative analyses were performed using sequences from seed plants in closely related taxa [7]–[9]. For example, 72 single-nucleotide polymorphisms (SNPs) and 27 indels were observed when two subspecies of rice (*Oryza sativa* spp. *indica* and *O. sativa* spp. *japonica*) were compared [7], and 32 SNPs were detected between the cp genomes of 17 *Jacobaea vulgaris* individuals [9]. The cp genomes of *Panicum virgatum*, which has different ecotypes in upland and lowland regions, contained 116 SNPs and 46 indels [10], and high variability in indel (3–278) and SNP (6–1000) numbers was noted in the cp genomes of 13 *Gossypium* species [11]. Finally, comparison of intraspecific variation in rare and widespread pines indicated low levels of divergence [8]. These data improved our understanding of cp genome evolution and divergence times of closely related taxa. Recent advances in rapid pyrosequencing techniques provide further opportunities to study population diversification and evolution using large numbers of whole cp genome sequences.

In monilophytes, a sister group to that of seed plants, no comparative cp genome analyses have been performed in intraspecific taxa despite the availability of seven complete cp genome sequences [12]–[15]. Previous monilophyte cp genome analyses focused on higher taxonomic levels and examined gene content, gene rearrangement, nucleotide substitution, and phylogenetic relationships.

Plant species distribution in different continents is a subject of ongoing interest to botanical researchers. The disjunctive distributions of similar flowering plant species in North America and East Asia have been extensively studied with respect to migration path, migration time, habitat similarity, and phylogenic relationship [16]–[23]. Single fern species are often distributed throughout the two continents. This contrasts with flowering plant distribution, in which the same species rarely occurs in both continents [24], [25]. One example is the *Adiantum pedatum* complex of leptosprangiate ferns: molecular data suggest that *A. pedatum* migrated from East Asia to North America through the Bering land-bridge and subsequently migrated from North America to East Asia [25]. Disjunctive distribution within a species is more easily observed in ferns than in flowering plants. Homosporous fern spores are easily dispersible and are able to live independently, facilitating the founding of a new population after migrations up to thousands of kilometers [26].

*E. arvense* is the most widely distributed fern globally, and is divided into two chemotypes. Plants in the European population do not contain flavonoids, but plants in the Asian and American populations contain luteolin 5-O-glucoside and malonyl ester [27]. However, morphological characteristics do not differ significantly between the three regional *E. arvense* populations and they are recognized as a single species.

With the exception of a common inversion in monilophytes, the American *E. arvense* cp genome structure in the large single-copy (LSC) region resembles the cp genome in moss and hornwort. The *trnY-trnE* intergenic spacer (IGS) region in the American *E. arvense* cp genome has a distinctive length of 5 kb, unlike in other monilophyte cp genomes. The lengths of *trnY-trnE* IGS usually less than I kb in other monilophytes [28]. This unusual *trnY-trnE* IGS length was also detected in *E. ramosissimum*, and this unique characteristic may be attributable to a repetition of the *trnY* anticodon loop [29].

In this study, we investigated the differences between the Asian and American *E. arvense* whole cp genomes. In addition, we analyzed repeat sequences in the *trnY-trnE* IGS region to determine the origin of the repeating unit that forms a hotspot in the cp genomes of genus *Equisetum*. Finally, we used the cp genome differences between Asian and American *E. arvense* to understand correlations between disjunctive distribution and cp genome divergence.

## Materials and Methods

### Chloroplast genome sequencing and annotation

*E. arvense* was collected from South Korea (H.-T. Kim, 2009-0413, voucher specimen in Korea University, Seoul (KUS), herbarium) and chloroplasts were separated from fresh leaves using a sucrose step-gradient method [30]. Cp DNAs (PDBK DNA No. 2009-0413) were isolated using 5× lysis buffer [31] and were sequenced using a Genome Sequencer FLX Titanium (Macrogen, Korea). Total FLX read numbers were 123,080, and the average read length was 337.5 bp. Six large contigs covered 90% of the total cp genome sequence. The *E. arvense* cp genome sequencing strategy was as indicated in Figure S1. Two additional PCR methods were used to fill the 10% gap and low-coverage regions (10%). A long-PCR method was used to fill gaps >3 kb, with conditions as follows: initial denaturation at 94°C, 4 min; (94°C, 15 s; 53–65°C, 30 s; 68°C, 3–10 min)×35 cycles; and post-extension at 72°C, 7 min. A short-PCR method was used to fill gaps <3 kb, with conditions as follows: initial denaturation at 94°C, 4 min; (94°C, 15 s; 50°C, 30 s; 72°C, 2 min)×35 cycles; and post-extension at 72°C, 3 min. PCR products were purified using column-based kits (Qiagen QIAquick PCR purification kit, Hilden, Germany) and sequenced using Big-Dye chemistry (Applied Biosystems, Foster City, CA, USA) and an ABI 3730XL sequencer.

The cp genome sequence of Asian *E. arvense* was assembled using Sequencher 4.7. Genes were annotated using DOGMA [32], and gene locations were determined by NCBI BLAST search (http://blast.ncbi.nlm.nih.gov/Blast.cgi). Secondary tRNA structures and location of tRNA genes were predicted using tRNAscan-SE 1.21 [33] Additional bioinformatic analyses were similar to those described in Kim and Lee [34], Lee et al. [3], and Yi and Kim [35].

### Comparison of complete cp genome sequences from Asian and American *E. arvense*

Indels and SNPs between the Asian and American *E. arvense* (NC_014699) cp genomes were detected as follows. The sequences of the two *E. arvense* cp genomes were divided into three functional segments (gene coding regions, introns, and intergenic spacers) and the partitioned sequences were aligned using the MUSCLE program [36]. Positions of indels and SNPs were determined using Geneious 5.6.5 [37]. Indels were divided into two types, A and B. Insertion was observed in the Asian *E. arvense* cp genome sequence for type A indels and in the American *E. arvense* cp genome for type B indels. Insertion event orientation was determined using *E. hyemale* and *Psilotum nudum* cp sequences as references for the outgroup sequence comparison method. A tandem-repeat finder [38] was used to classify indels according to location in 1) mononucleotide repeat regions, 2) tandem-repeat regions, and 3) dispersed repeat regions. The folding structure of the *rrn16* gene was predicted using the Mfold Web Server [39]. The *rrn16* gene sequences from three eusporangiate ferns and *Osmunda cinnamomea* were obtained from GenBank (KF 225592, NC 008829, NC 017006, and NC 003386) and were used for folding structure comparisons.

### Analysis of *trnY*-*trnE* IGS sequences between *Equisetum* species

Six sequences representing the two *Equisetum* subgenera [40], [41] were used to analyze differences between species in the *trnY-trnE* IGS region. Four sequences were obtained from GenBank and two were generated in this study (Table 1). *E. hyemale* was collected in South Korea (H.-W. Kim 2007-0543, voucher specimen in KUS herbarium) and genomic DNA (PDBK DNA No. 2007-0543, voucher specimen in KUS herbarium) was extracted from fresh leaves using the CTAB method [42]. DNA was purified using cesium chloroide/ethidium bromide gradients. Primers were designed to amplify and sequence the *trnY*-*trnE* IGS region of *E. hyemale* (Forward primer: CAAAGCCAGCGGATTTACAA, Reverse primer: CCCCATCGTCTAGTGGCCTA) using the cp genome sequence of *E. arvense* as a reference sequence. The long-PCR method was used to amplify the region. Sequences were assembled with Sequencher 4.7 and the locations of the *trnY* and *trnE* genes were confirmed using DOGMA. The six *trnY-trnE* IGS sequences were aligned using the MUSCLE alignment program [36].

**Table 1:** The list of taxa used in this study.

Location	Subgenus	Species	GenBank accession #
America	*Hippochaete*	*Equisetum hyemale*	KC117177^a^
Asia(Korea)		*Equisetum hyemale*	KC610090^b^
Asia(China)		*Equisetum ramosissimum*	HQ658109^b^
America	*Equisetum*	*Equisetum arvense*	NC014699^a^
Asia(Korea)		*Equisetum arvense*	JN968380^a^
Asia(china)		*Equisetum arvense*	HQ658110^b^

a Complete cp genome.

b The trnY-trnE region sequence.

The GenBank accession numbers KC610090 and JN968380 were reported in this study and all other sequences are obtained from GenBank.

Repeat sequences in *trnY*-*trnE* IGS were analyzed using REPuter [43] with a 10 bp minimum length of repeat sequence and a Hamming value of 3 bp. Repeat sequence frequencies were detected using DNA Pattern Search (http://www.geneinfinity.org/sms/sms_DNApatterns.html#). The formation of repeat sequence hairpin structures was confirmed using the Mfold Web Server [39]. Consensus repeat sequences were numbered by position and sequence frequencies at each position were also calculated. Dot-matrix analysis (Serolis dot-plot software version 0.9.9, available from http://www.code10.info) was used to assess the distribution patterns of repeated units and the conservation levels of each repeat unit in the *trnY-trnE* IGS region.

## Results

### Length variation caused by insertions/deletions in two *E. arvense* cp genomes

**Figure 1:**
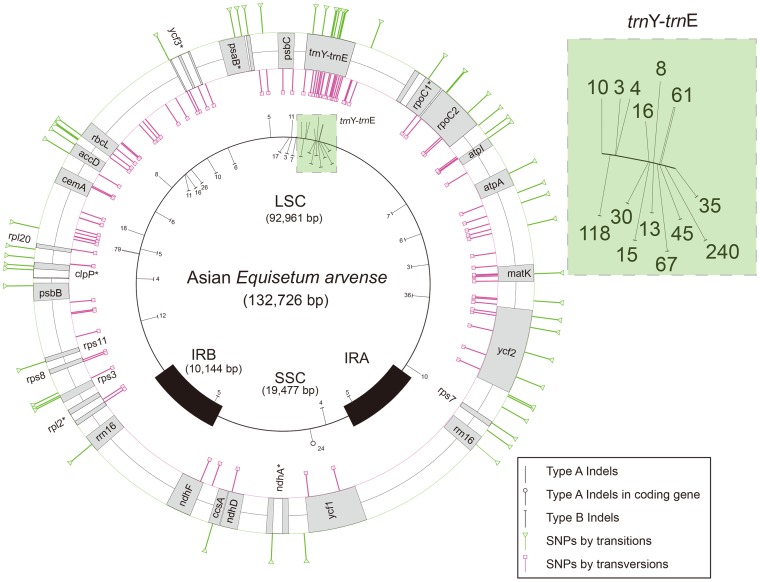
Distribution map of indels and SNPs on the cp genomes of *Equisetum arvense*. This map was generated by comparing Asian and American *E. arvense* individuals. Indels ≥3 bp in length are indicated on the inner circle and SNPs are indicated on the outer circle. Genes with SNP(s) are listed on the outer circle. The numeric values on the inner circle indicate the indel length at each location. A gene name annotated with an asterisk indicates an intron-containing gene. Detailed locations of SNPs on relevant gene(s) and IGS region(s) are marked on the outer circle. The green box at the right upper corner shows indel locations and indel length on the *trnY-trnE* IGS region.

**Figure 2:**
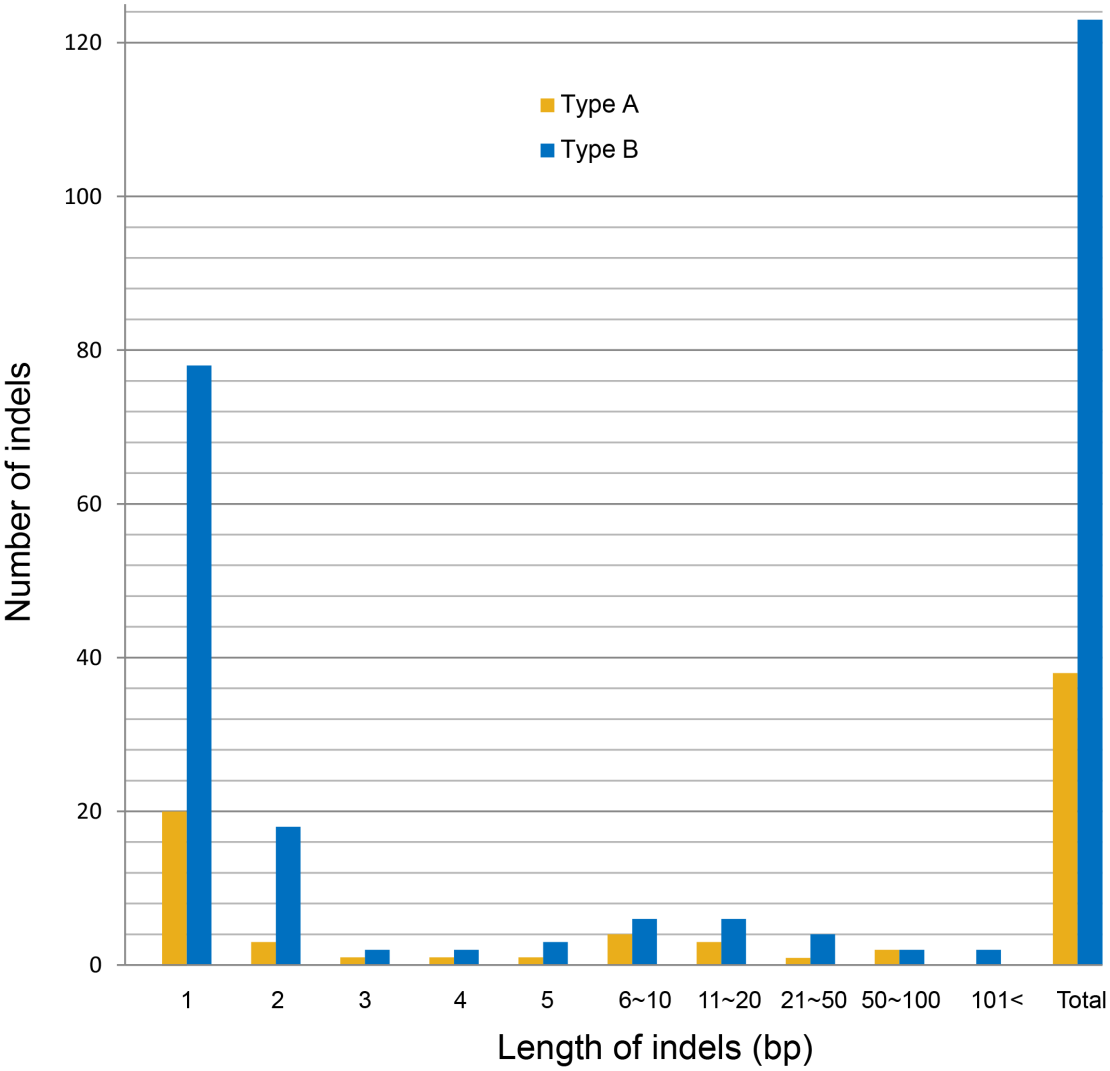
Bar graph showing the number (Y axis) and length (X axis) of indels. Type B indels were predominant over type A indels (see text).

The complete cp genome sequence of Asian *E. arvense* was 132,726 bp in length, comprising a 92,961 bp LSC region, a 19,477 bp small single-copy (SSC) region, and two 10,144 bp inverted repeats (IRs) (Fig. 1). Comparison of the Asian and American *E. arvense* cp genomes indicated that the Asian *E. arvense* cp genome LSC region was 581 bp shorter, the IR region 5 bp shorter, and the SSC region 8 bp longer than in the American *E. arvense* cp genome (Table 2). Most of the 159 indels (69.2%) occurred in homopolymer regions, and most were type B indels. The remaining indels occurred in tandem-repeat regions and were more than 2 bp in length (15.1%) or were in non-repeat regions (15.7%). Regionally, 142 indels (89.3%) were detected in the LSC region, 13 (8.2%) were detected in the SSC region, and four (2.5%) were detected in the IR regions. The indel number per unit length ratio was 7.7∶3.4∶1 (LSC∶SSC∶IR). All indels were found in noncoding regions with the exception of an indel affecting the *ycf1* gene in the SSC region. An insertion of a 24 bp (ATCAATGCTAGATGTTTCAAAAGT) tandem-repeat unit was observed in the *ycf1* gene in Asian *E. arvense*. Most indels (75%) were 1–2 bp in length; these accounted for 15% of the difference in length between the Asian and American *E. arvense* cp genomes (Fig. 2). Indels ≥3 bp long accounted for 85% of the length difference between the two genomes. The ≥3 bp indels (13 type A and 27 type B) were found throughout the cp genome. A high proportion (35.9%) were concentrated in the *trnY*-*trnE* IGS region (Fig. 1), with the remainder evenly distributed across the rest of the cp genome.

**Table 2:** Indel events on the cp genomes of Asian and American *E. arvense*.

	LSC		SSC		IR(×2)		Total	
	Type A	Type B	Type A	Type B	Type A	Type B	Type A	Type B
Indels on homopolymer regions	17	80	1	10	0	1	18	92
Indels on tandem repeat regions	6	15	1	0	0	1	7	17
Indels on other regions	11	13	0	1	0	0	11	14
**total**	34	108	2	11	0	4	36	123

### Single-Nucleotide Polymorphisms in two *E. arvense* cp genomes

**Figure 3:**
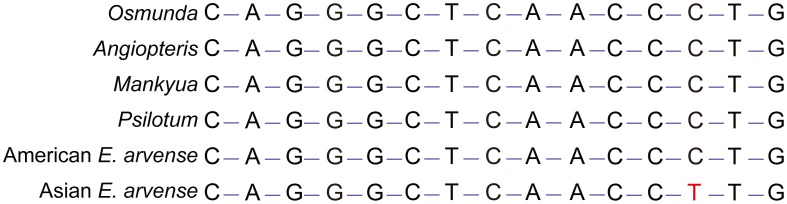
Aligned SNP sequences of the *rrn16* gene region. All eusporangiate ferns and *Osmunda cinnamomea*, except Asian *E. arvense*, contained identical sequences. C to T substitution was observed in Asian *E. arvense*. Ten *E. arvense* individuals from various locations in Korea, Japan, and China share the C to T substitution.

In total, 165 SNPs were detected between the Asian and American *E. arvense* genomes (Fig. 1). Of these, 155 (93.9%) were found in the LSC region, eight (4.8%) were located in the SSC region, and two (1.2%) were found in the IR regions (Table 3). The SNP number per unit length ratio was 16.8∶4.2∶1 (LSC∶SSC∶IR). Most SNPs (100; 60.6%) were found in IGS, 50 SNPs (30.5%) were located in protein-coding genes, 13 SNPs (7.9%) were in introns, and two SNPs (1.2%) were found in an rRNA gene. Thirty-five SNPs were concentrated in the *trnY*-*trnE* IGS region. The SNP in the IR region was detected in the *rrn16* gene, and was a unique base substitution reported only in Asian *E. arvense* (Fig. 3). The putative folding structures of *rrn16* due to the SNP are compared in the Figure S2. Of the 50 SNPs found in protein-coding genes, 23 were transitional changes (Ts) and 27 were transversional changes (Tv) (Table 4). Twenty Ts and ten Tv changes were synonymous substitutions (Ks), and three Ts and seven Tv changes were nonsynonymous (Kn). SNPs were detected in 26 of the 84 protein-coding genes. The highest K2P distance was observed for the *matK* gene, which had five Kn changes. The most substitutions were found in the *ycf2* gene, with five Ks and four Kn changes. The Ts/Tv ratio for the whole cp genome was 0.53, while the Ts/Tv ratio for the coding region alone was 0.85. The Ts/Tv ratio in coding regions (0.85) was two-fold higher than the ratio in IGS regions (0.39) and introns (0.41).

**Table 3:** SNPs on the cp genomes of Asian and American *E. arvense*.

	Coding regions		IGSs		Introns		rRNAs		Total	
	Ts	Tv	Ts	Tv	Ts	Tv	Ts	Tv	Ts	Tv
**LSC**	21	23	28	71	4	9	0	0	53	103
**SSC**	2	4	0	1	0	0	0	0	2	5
**IR(×2)**	0	0	0	0	0	0	1	0	2	0
**Total**	23	27	28	72	4	9	2	0	57	108

Ts and Tv indicate transitional and transversional changes, respectively.

**Table 4:** Distribution of nucleotide substitutions on coding genes of the cp genomes of Asian and American *E. arvense* populations.

Region	Gene	Ts	Tv	S	N
**LSC (21 genes)**	*accD*	1	0	1	0
	*atpA*	1	0	1	0
	*atpI*	1	0	0	1
	*cemA*	0	3	1	2
	*clpP*	1	0	1	0
	*matK*	1	4	0	5
	*psaB*	2	0	2	0
	*psbB*	1	0	1	0
	*psbC*	0	1	1	0
	*rbcL*	1	2	2	1
	*rpl2*	0	1	0	1
	*rpl20*	0	1	0	1
	*rpoC1*	1	1	1	1
	*rpoC2*	3	3	4	2
	*rps11*	1	0	1	0
	*rps14*	1	0	1	0
	*rps3*	1	0	1	0
	*rps7*	1	0	1	0
	*rps8*	0	1	0	1
	*ycf2*	4	5	5	4
	*ycf3*	0	1	1	0
**SSC (5 genes)**	*ccsA*	1	0	1	0
	*ndhA*	1	0	1	0
	*ndhD*	0	1	0	1
	*ndhF*	0	1	1	0
	*ycf1*	0	2	2	0
**Total**		23	27	30	20

### Hairpin structures in the *trnY*-*trnE* IGS in genus *Equisetum*

The *trnY*-*trnE* IGS was 4,534 bp and 4,991 bp in length in Asian and American *E. arvense*, respectively. This region comprised only 3.4% of the genome but was responsible for 78% of the total variation in length between the two cp genomes. Several secondary-structure-forming repeats were located in the *trnY*-*trnE* IGS region and had a 19 bp basic unit.

**Figure 4:**
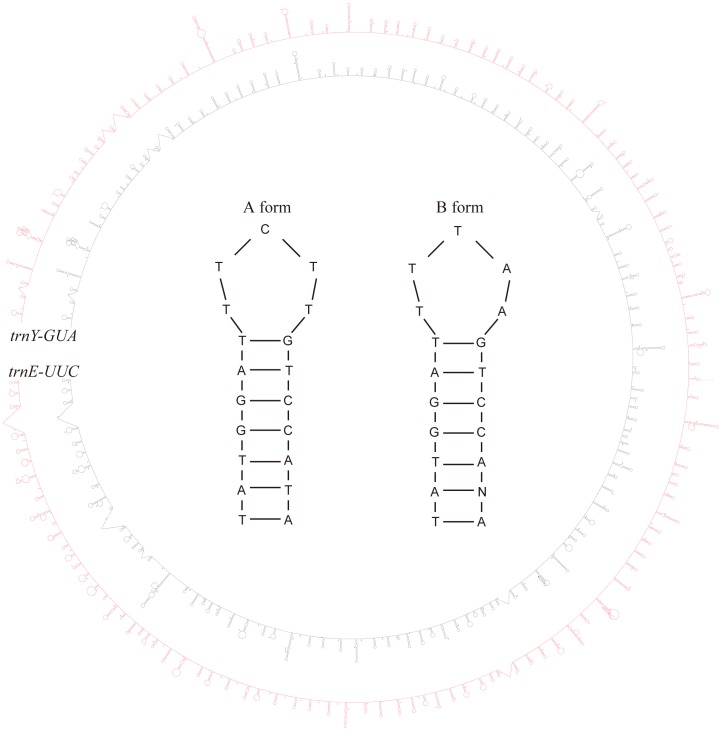
Putative folding structures of *trnY*-*trnE* IGS sequences in Asian and American *E. arvense*. The inner circle indicates Asian *E. arvense* sequence and the outer circle indicates American *E. arvense* sequence. Both sequences had numerous hairpin structures that were largely distributed evenly across the entire region. The most common two hairpin sequences (A and B forms) are shown inside the two circles. The two forms differed mainly on the loop region sequences. The most frequent base at the N position in the stem of the B form was G or T followed by A or C.

The number of repeats differed between the genomes, with 66 in Asian *E. arvense* and 73 in American *E. arvense*. Most of the repeating units were in one of two different sequence forms, A and B. The A-form consisted of a 7 bp stem region (TATGGAT) and a 5 bp loop region (TTCTT). The same stem region (TATGGAT) was observed in the B-form, but a different loop region was present (TTTAA) (Fig. 4). Eighteen A-form and twenty-three B-form repeats were detected in Asian *E. arvense*, while twenty-eight A-form and twenty-eight B-form repeats were detected in American *E. arvnese*. Indels greater than 10 bp in length occurred mainly in the stem regions of these hairpin structures and contributed to the variation in length between the Asian and American *E. arvense* sequences. In addition, two indels were detected between Chinese and Korean individuals of Asian *E. arvense*, both of which were associated with the A-form repeating unit.

**Figure 5:**
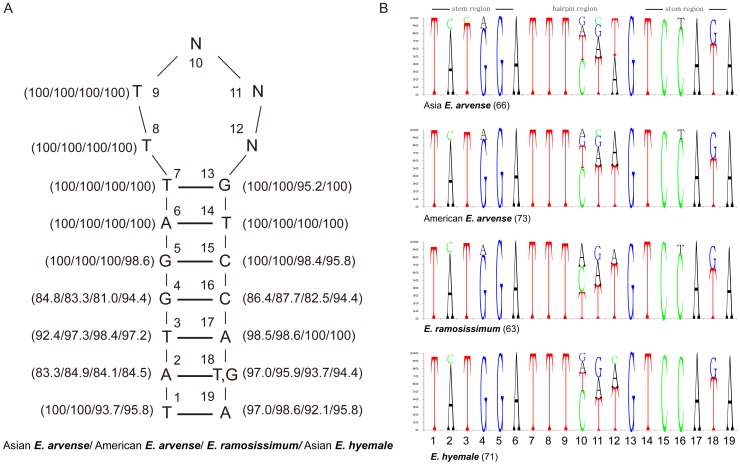
Consensus sequences for the repeated hairpin structure in the *trnY*-*trnE* IGS region. (A) The most common consensus 19 bp sequences and frequencies were as indicated on the hairpin structure. Positions 6, 7, 8, 9, and 14 were invariable and positions 10, 11, and 12 were the most variable sites. (B) Consensus sequences were derived from the 66 repeats of Asian *E. arvense*, the 73 repeats of American *E. arvense*, the 63 repeats of *E. ramosissimum*, and the 71 repeats of *E. hyemale*.

Other species from genus *Equisetum* varied in the length of their *trnY*-*trnE* IGS region. These species had similar 19 bp repeating units to those in *E. arvense*, but sequence units were slightly modified (Fig. 5). *E. hyemale* and *E. ramosissimum* had 71 and 63 of the 19 bp repeat units, respectively. These repeating units were highly conserved both in structure and sequence, with a higher level of variation observed in the loop region than the stem region (Fig. 5). Stem region sequences were identical at the positions from 5 to 9 in all individual units with the exception of a single A-T pairing of the 5th–15th bases in *E. hyemale*. In addition, the 13th, 14th, and 15th bases paired with the 7th, 6th, and 5th bases, respectively, and showed over 95% identity between/among the four species. The 1st–19th and 17th–3rd pairings showed greater than 90% identity between/among the four species.

The 2nd base was most frequently A (83–85%), while the 18th position was usually T (63–69%). C was occasionally found at the 2nd base position (11–12%), and G was sometimes at the 18th base position (25–32%). Three main base-pairings were observed between the 2nd and 18th base positions: A-T had the highest frequency, followed by A-G and C-G pairs. The 10th, 11th, and 12th bases were hypervariable sites, and the main nucleotides at these positions varied according to species. For example, CTT was the main sequence in subgenus *Equisetum*. In subgenus *Hippochaete*, however, ATT was predominant in *E. ramosissimum* but CTT was the main sequence found in *E. hyemale*.

The total sequence lengths in the two species from the subgenus *Hippochaete* were longer than the sequences from two *E. arvnese* of subgenus *Equisetum*. We calculated the p-distance and the number of differences in the *trnY*-*trnE* IGSs between the species (Table 5). The intraspecific p-distances between *E. hyemale* and the two continental individuals of *E. arvense* were both 0.008. The distance between the two Asian individuals was 0.0004. The interspecific p-distances in subgenus *Hippochaete* (*E. hyemale* and *E. ramosissimum*) were in the range 0.031–0.034. The interspecific p-distances between the two subgenera (subgenus *Hippochaete* and subgenus *Equisetum*) were in the range 0.084–0.096. The lowest interspecific p-distance value was observed between Korean *E. arvense* and American *E. hyemale* and the highest value was seen between *E. ramosissimum* and American *E. arvense*.

**Table 5:** No. of nucleotide differences and p-distances of the *trnY*-*trnE* IGS region in genus *Equisetum*.

	Korean *E. arvense*	Chinese *E. arvense*	American *E. arvense*	Korean *E. hyemale*	American *E. hyemale*	E. *ramosissimum*
**Korean ** ***E. arvense*** ** (4534 bp)**	-	2	35	351	333	360
**Chinese ** ***E. arvense*** ** (4872 bp)**	0.0004	-	38	378	369	375
**American ** ***E. arvense*** ** (4991 bp)**	0.008	0.008	-	397	356	385
**Korean ** ***E. hyemale*** ** (5400 bp)**	0.090	0.092	0.095	-	44	147
**American ** ***E. hyemale*** ** (5645 bp)**	0.084	0.089	0.084	0.008	-	159
***E. ramosissimum*** ** (5000 bp)**	0.095	0.095	0.096	0.031	0.034	-

**Figure 6:**
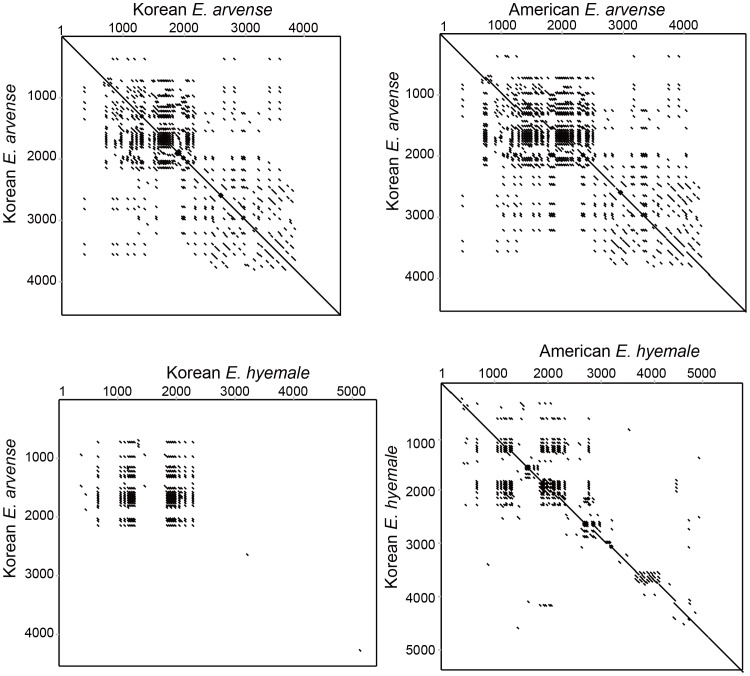
Dot matrix showing the occurrence, conservation, and divergence of the repeating units in the *trnY*-*trnE* IGS region. The region proximal to the *trnY* gene (1–2,200 bp region) had high density and conservation of repeating sequences compared to the region proximal to the *trnE* gene (>2,200 bp region).

Dot-plot analysis of Korean *E. arvense* indicated that repeating units were widespread and concentrated in the first half of the *trnY-trnE* IGS region (Fig. 6). A similar pattern was observed with dot-plot analysis of Asian and American *E. arvense*. A few repeat units were found in the second half of the *trnY-trnE* IGS region in *E. arvense* and *E. hyemale. E. arvense* and *E. hyemale* had many similar repeat units, and these were located primarily in the 800 to 2,200 bp region. Overall, repeat unit conservation was more prominent in the first half of the IGS than the second half.

## Discussion

### Molecular evolution of the cp genome in Asian and American *E. arvense*

The Asian and American *E. arvense* cp genomes were of different lengths, and this variation was due to the presence of 159 indels. The majority of the indels were homopolymer length variants; however, these can be produced as artifacts of the 454-sequencing process [44], [45]. Nevertheless, we were confident of the accuracy of our complete sequences for three reasons. First, our pyrosequencing contigs covered the majority of specific sequences with 100× coverage and the majority consensus sequences were derived from numerous overlapping contigs. Second, we manually amplified and sequenced low-coverage regions and found many homo- and heteropolymer repeating regions. Third, the cp genome of the *Olea europaea* complex had approximately 4–8× more 1–2 bp indels than >3 bp indels [46], whereas the ratio of 1–2 bp to >3 bp indels in *E. arvense* was 3∶1. If the *E. arvense* indels were identified mainly as a result of 454-pyrosequencing errors, the indel ratio would be biased towards 1–2 bp rather than >3 bp indels. Therefore, we believe that the majority of the homopolymer indels found in *E. arvense* were true indels that reflected the evolutionary history of the two continental individuals.

Relatively few large indels (>3 bp) were previously observed in closely related taxa of plant genera. For example, only seven indels were reported in the cp genomes of the *O. europaea* complex. Nine indels were observed in three species of *Phyllostachys* and 3–21 indels were reported in *Gossypium* species. No indels were detected between the two cp genomes of *Nicotiana tabacum* and *N. sylvestis*. By contrast, 40 indels of >3 bp were seen in Asian and American *E. arvense* despite the recognition of these two continental populations as a single species. In this study, we excluded 14 indels that were identified from the hypervariable *trnY*-*trnE* region of the *Equisetum* cp genome. Therefore, our data clearly indicated that the variation between the cp genomes of the two *E. arvense* was much higher than the intersubspecific or the interspecific differences observed in various flowering plants. The high intraspecific difference observed between the two continental *E. arvense* was also confirmed by SNP analysis.

SNP variation between Asian and American *E. arvense* was 0.12%. This variation level was higher than intraspecific or interspecific differences previously reported in numerous seed plants, with intraspecific SNP variation being very low in many plant groups. For example, SNP variation was 0.07% between two interecotypes of *Panicum virgatum* [10], and was 0.02% between 17 individuals of *Jacobaea vulgaris* [9]. In addition, SNP variation was 0.05% between *Oryza sativa* subsp. *indica* and *O. sativa* subsp. *japonica* [7] and 0.03–0.07% in the *Olea europaea* complex [46]. The SNP variation between *E. arvense* individuals was higher than interspecific variation observed in several cases. Variation was 0.003% between *Nicotiana tabacum* and *N. sylvestis*, 0.007–0.11% between five *Gossypium* species [11], and 0.02–0.05% between three *Phyllostachys* species [47]. In addition to the substantial indel and SNP differences between Asian and American *E. arvense*, the genetic distance between the two *E. arvense* individuals was higher than the interspecific variation in flowering plants. The high genetic diversity and low morphological diversity indicated that the two continental *E. arvense* populations had high genetic heterogeneity while being in morphological stasis.

A few examples of regionally isolated flowering plant species in similar habitats exhibiting similar morphological characteristics have been reported [19], [48]. The two continental populations of *E. arvense* experience similar habitats and have no morphological differences, yet are geographically isolated in Asia and North America. This suggested that the cp genomes would differ considerably as the two *E. arvense* populations were split by disjunctive distribution. The high variance between the cp genomes of the two continental *E. arvense* suggests relatively ancestral divergence if a molecular clock concept is applied.

To estimate the divergence time between Asian and American *E. arvense*, we adopted different SNP data from five data sets. The whole cp SNP data between the two inter-subspecies of *Oryza sativa* (ssp. *indica* and ssp. *japonica*) and two ecotypes of *P. virgatum* were used. The divergence time of the two *O. sativa* inter-subspecies was estimated at 0.4 mya [50], and the divergence time of the two *P. virgatum* eocotypes was estimated at 0.7–1.0 mya [51]. SNP variation between the two *E. arvense* was 2.3× higher than between the two *O. sativa* inter-subspecies and 1.5× higher than between the two *P. virgatum* eocotypes. This suggested that the two *E. arvense* populations diverged 1.0–1.7 mya. We also estimated the divergence time of the two *E. arvense* populations using the partial and whole cp SNP data from the same *Equisetum* genus using a published calibration clock [49]. There are three different data sets available from *Equisetum*. First, the *rbcL* calibration clock suggested that subgenus *Equisetum* and subgenus *Hypochaete* diverged approximately 28.5±5.5 mya. Differences in *rbcL* between the two *E. arvense* were 15× lower than between two subgenera of *Equisetum*. Therefore, the *rbcL* calibration clock suggested that the two *E. arvense* populations diverged approximately 1.9±0.4 mya. Second, the hypervariable *trnY-trnE* intergenic spacer different between the two subgenus *Equisetum* and subgenus *Hypochaete* was 9.1% (Table 5) and they diverged approximately 28.5±5.5 mya. Differences in the hypervariable *trnY-trnE* intergenic spacer between the two *E. arvense* were 11.4× lower than between two subgenera of *Equisetum*. Therefore, the hypervariable *trnY-trnE* intergenic spacer data suggested that the two *E. arvense* populations diverged approximately 2.5±0.5 mya. Third, the cp SNP difference between the subgenus *Equisetum (E. arvense)* and subgenus *Hypochaete (E. hyemale)* was 1.44% and they diverged approximately 28.5±5.5 mya as in the *rbcL* calibration. Differences in the whole cp SNP between the two *E. arvense* were 12× lower than between two subgenera of *Equisetum*. Therefore, the whole cp SNP calibration suggested that the two *E. arvense* populations diverged approximately 2.4±0.5 mya. The five independent estimates of divergence time are relatively concordant and fall into an overlapping range. However, we are more confident to the *Equisetum* calibration than the Poaceae calibration because of two reasons. First, the *Equisetum* clock is based upon abundant fossil records. Second, the *Equisetum* calibration uses data from the same lineages, minimizing the lineage bias effect of the molecular clock.

Phylogenetic analysis of genus *Equisetum* estimated the speciation time of *E. arvense* to be 2.588 mya, which lies between the Pliocene and Quaternary periods [49]. *E. arvense* may have migrated from one region to another through the Bering land-bridge after speciation, in a similar manner to the migration that produced the Asian and North American *Adiantum pedatum* complexes. Japanese *A. pedatum* and northeastern American *A. pedatum* diverged 2.47 mya. Northeastern American *A. pedatum* and Chinese *A. pedatum* subsequently diverged 1.09 mya [25]. The disjunctive distribution of northeastern American *A. pedatum* and Chinese *A. pedatum* through the Bering land-bridge in the mid-Pleistocene is very similar to the scenario for the disjunctive distribution of Asian and American *E. arvense*. Therefore, we believe that *E. arvense* and *A. pedatum* might have migrated during the same period, stimulated by the geological or environmental conditions at that time. The molecular differences therefore accumulated between the two continental *E. arvense* populations since the mid-Pleistocene, 1.9–2.9 mya, with almost no corresponding development of morphological differences.

One rRNA SNP in Asian *E. arvense* was of particular interest because it was shared by all the Asian individuals, suggesting a single origin for all the Asian individuals. *E. arvense* is a member of the eusporangiate ferns, which is the basal ferns lineage. Other eusporangiate ferns (*Mankyua chejuense*, *Psilotum nudum* and *Angiopteris evecta*) and basal leptosporangiate fern (*Osmunda cinamomea*) share the same rRNA sequence as American *E. arvense* (Fig. 3B). The CCCUG sequence in the *rrn16* gene of eusporangiate ferns, including American *E. arvense* and *O. cinamomea*, produces a hairpin structure by pairing with CAGGG (Fig. 3). However, in the *rrn16* gene of Asian *E. arvense*, the CCCUG sequence was mutated (to CCUUG) and the CCUUG sequence was paired with CAGGG. Therefore, the *rrn16* gene of Asian *E. arvense* had a less stable folding structure than the equivalent American *E. arvense* sequence (Figure S2). The SNP in the *rrn16* gene therefore changed the minimum free energy for RNA secondary structure. We tested for this SNP using PCR-sequencing techniques in ten Asian *E. arvense* individuals from Korea, Japan, and China. All Asian individuals shared this SNP.

### Repeat sequence evolution in genus *Equisetum*

Eusporangiate ferns consist of four orders, including Equisetales [52], and the cp genome of one or two species from each order has been sequenced [13], [28], [53]. However, the *trnY*-*trnE* IGS expanded up to 5 kb in *Equisetum* and the duplication of a 19 bp repeating unit was responsible for this expansion. The 19 bp repeating unit was not detected in the cp genomes of the other three orders. This indicated that the 19 bp repeating unit might be a unique molecular characteristic that only occurred in the monotypic genus *Equisetum*. The genus *Equisetum* is divided into two subgenera and the 19 bp repeating units with hairpin structures were identified in both. The sequence identity of the 19 bp unit was sufficiently conserved to allow easy recognition. We therefore assumed that the consensus repeating unit originated prior to the separation of the two subgenera in genus *Equisetum*. Similar expansion of the *trnY*-*trnE* IGS (albeit shorter; ∼450 bp) was reported in the leptosporangiate fern *Vanderboschia radicans*. In that case, duplication of a 27 bp repeat unit homologous to a *trnY* anticodon was responsible for the expansion of the *trnY*-*trnE* IGS [29]. A similar duplicated expansion of *trnD*-*trnY* IGS was responsible for length variation in *Pseudotsuga* species (Gymnosperms) [54]. We compared the *Equisetum trnY*-*trnE* IGS repeat unit to those in other species but were unable to conclusively determine the origin of the 19 bp repeat unit in *Equisetum trnY*-*trnE* IGS as sequence identity with *trnY* was low. Gao et al. [29] suggested that a 13 bp repeat in the *Equisetum trnY*-*trnE* IGS may be derived from partial *trnY* sequences. However, our data do not support this as the extended stem region in our 19 bp consensus repeat unit is substantially different from *trnY* anticodon sequences. Several cp *trn* genes hold similar sequence components and it is therefore difficult to deduce the origin of the *trn* repeat unit. Furthermore, the *trnY* duplication occurs at slightly different locations in *Pseudotsuga* and *V. radicans*. *Pseudotsuga*, *Equisetum*, and *V. radicans* are not phylogenetically close and no known *trnY*-*trnE* IGS expansions have occurred in the sister groups of these three lineages. Therefore, we believe that the *trnY*-*trnE* IGS expansions in *Pseudotsuga*, *Equisetum* and *V. radicans* were independent parallel evolutionary events rather than a homologous synapomorphy. By contrast, the *trnY*-*trnE* IGS expansion was discovered in all *Equisetum* species examined in this study and is a single evolutionary event.

Fossils of the genus *Equisetites*, which is the most closely related genera to genus *Equisetum* [55], were discovered worldwide in Europe [56], North America [57], Antarctica [58], China [59], and New Zealand [60] in underlying strata of the post-Mesozoic era. This suggested that the divergence time for genus *Equisetum* was in the Tertiary period. Extant *Equisetum* species diverged more recently than the Miocene period [49]. Therefore, we reasoned that the repeating units were formed during the Miocene period after or before the formation of genus *Equisetum*, and the repeating units then spread widely and diverged alongside *Equisetum* speciation events. To summarize, the 19 bp repeating unit is a synapomorphic molecular characteristic shared by all living members of *Equisetum*.

The *trnY*-*trnE* IGS region differed in length between Korean and Chinese *E. arvense* individuals. Chinese *E. arvense* had 76 repeat sequences and a *trnY-trnE* IGS length of 4,872 bp. When repeat sequence numbers and *trnY*-*trnE* IGS lengths were compared, Chinese *E. arvense* was more superficially similar to American *E. arvense* than to Korean *E. arvense*. However, when the indel and SNP patterns were considered together, Chinese *E. arvense* was found to be more closely related to Korean *E. arvense* than to American *E. arvense*. Two large indels (241 bp, 97 bp) and two SNPs were detected between Korean *E. arvense* and Chinese *E. arvense*, but 19 indels and 38 SNP differences were noted between the Chinese and American *E. arvense* individuals.

The difference in the number of repeating units observed in Korean *E. arvense* and Chinese *E. arvense* suggested unequal crossover on the tandem-repeat regions. Two unequal crossovers may have generated the large length difference between the Korean and Chinese *E. arvense* individuals. The two large indels, composed of 241 bp and 97 bp, contained six and three 19 bp repeating units, respectively. Small indels of 1–10 bp were not found between Korean and Chinese *E. arvense*. In contrast to the large indels, the large numbers of small indels observed between Korean and American *E. arvense* suggested that the majority of small indels originated as mutation events formed by slipped strand mispairing [61]. A few indels with long length differences were observed between Korean and American *E. arvense*.

Analysis of the 19 bp repeat sequence (TATGGATTTCTTGTCCATA) (Fig. 5), suggested that the original source sequence was the *trnY*-*trnE* IGS. The 19 bp sequence found in this study is 6 bp longer than the *trnY* anticodon partial sequence proposed as the repeating unit origin [29]. Additionally, the 19 bp sequence had higher similarities than the 13 bp consensus sequence of the *trnY* anticodon loop region suggested in previous studies [29]. Our 19 bp consensus sequence was based on the wide range of sequence data available for the diverse *Equisetum* taxa. The expected origin sequence of repeating units was commonly abundant not only in subgenus *Equisetum* but also in subgenus *Hippochaete*. In *E. ramosissimum*, of subgenus *Hippochaete*, the repeating unit of TATGGATTTATTGTCCATA, which differed from the *E arvense* consensus at the 10th position (C to A), was found at a slightly higher frequency than the TATGGATTTCTTGTCCATA sequence.

The 11th and 12th sites, located in the hairpin loop region, showed A to T substitutions. Substitutions, albeit rare, were also observed at the 2nd, 4th, 16th, and 18th sites of the stem region. This indicated that the 19 bp repeating units have mutated constantly and maintained evolutionary heterogeneity in the genus *Equisetum*. Heterogeneity of the repeating unit was confirmed by dot-plot analysis (Fig. 6). The first half of the *trnY*-*trnE* IGS had more conserved repeating units than the second half. When compared with the length of the *trnY*-*trnE* IGS in other eusporangiate ferns, it was apparent that many repeats occurred in the second half of *trnY*-*trnE* IGS, but no apparent homologous repeating sequences were observed between *E. arvense* and *E. hyemale*. Therefore, we assumed that the progression to heterogeneity in the repeating unit proceeded particularly rapidly in the second half of the *trnY*-*trnE* IGS.

## Conclusions

Molecular clock analysis suggested that *E. arvense* migrated between two continents via the Bering land-bridge 1.9–2.9 mya. After migration, its morphological characteristics remained largely unchanged in each region due to adaption to similar habitats, but constant mutational events occurred in the cp genomes. This indicated that the two continental populations of *E. arvense* have been in prolonged morphological stasis while the cp genome sequences in the two regions have changed continuously since population dispersal. The levels of sequence and indel divergence between the two regional cp genomes were far higher than those of closely related interspecific taxa in many seed plants. Two regional genotypes can therefore be recognized. Rigorous comparative analyses of the whole cp genomes from multiple accessions of each continental population, including European populations, are needed to comprehensively address population history and the validity of the species boundary.

The *trnY*-*trnE* IGS is a hypervariable region within the *E. arvense* cp genome, and many indel events and SNPs were concentrated in this region. A unique 19 bp repeating sequence unit that formed a hairpin structure was replicated many times in this IGS region and was responsible for the dynamic sequence evolution of the cp genome. The genus *Equisetum* is a monotypic genus in Equisetales and the repeating units did not exist in other eusporangiate ferns. It was therefore challenging to find the exact origin sequence and to explain the evolutionary paths of repeat unit evolution. A comprehensive study involving additional *Equisetum* species is needed to understand the evolution of repeat units in the genus. However, with the current limited data set, the region showed very different intraspecific, interspecific, and intersubgeneric p-distance values. Therefore, the hypervariable *trnY*-*trnE* IGS region may be a useful molecular marker to study the evolution and phylogeny of *Equisetum*.

## Supporting Information

Figure S1 Sequencing strategy for the *E. arvense* chloroplast genome. The outer blue circle indicates the sequence region generated by next –generation sequencing (NGS). Seven large NGS contigs cover approximately 90% of the genome. The green broken lines indicate the regions sequenced by PCR amplifications and Sanger sequencing. The *trnY-trnE* IGS was amplified by long-range PCR methods. The genome map in the inner circle was generated in OrganellarGenomeDRAW [62] after the completion of sequencing and annotation.
(TIF)

Figure S2 The RNA folding structure differences of *rrn16* gene from two *E. arvense* populations. The gray box regions indicated the folding structure differences due to the SNP(C-U). The sequence -CCCUG- paired with the sequence –CAGGG- and form a hairpin structure in the American *E. arvense* (left). However, the sequence –CCUUG- paired with the sequence –CAAGG- and form a distinct stem structure in the Korean *E. arvense* (left). Two contrasting folding structures are based on the minimum free energy only. Other alternate folding structures are also possible if we consider other factors affecting the secondary structures.
(TIF)
