# Supplementary figures and images for: Chloroplast Genome Differences between Asian and American Equisetum arvense (Equisetaceae) and the Origin of the Hypervariable trnY-trnE Intergenic Spacer
Source: PLoS One. 2014 Aug 26;9(8):e103898. doi: 10.1371/journal.pone.0103898 (PMC4144802; doi:10.1371/journal.pone.0103898)

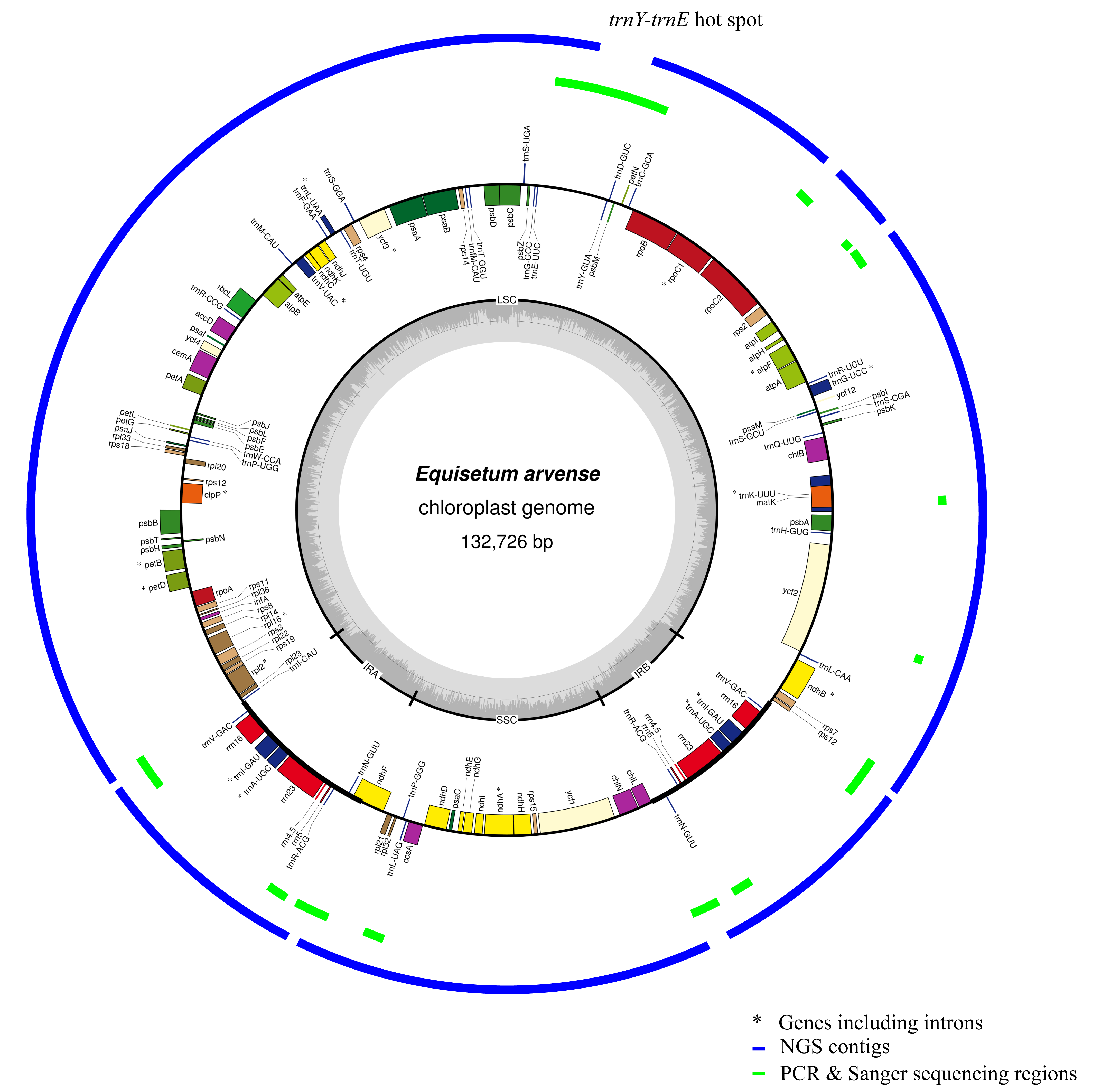

Supplement: Figure S1 — Sequencing strategy for the E. arvense chloroplast genome. The outer blue circle indicates the sequence region generated by next –generation sequencing (NGS). Seven large NGS contigs cover approximately 90% of the genome. The green broken lines indicate the regions sequenced by PCR amplifications and Sanger sequencing. The trnY-trnE IGS was amplified by long-range PCR methods. The genome map in the inner circle was generated in OrganellarGenomeDRAW [62] after the completion of sequencing and annotation. (TIF) [file pone.0103898.s001.tif]

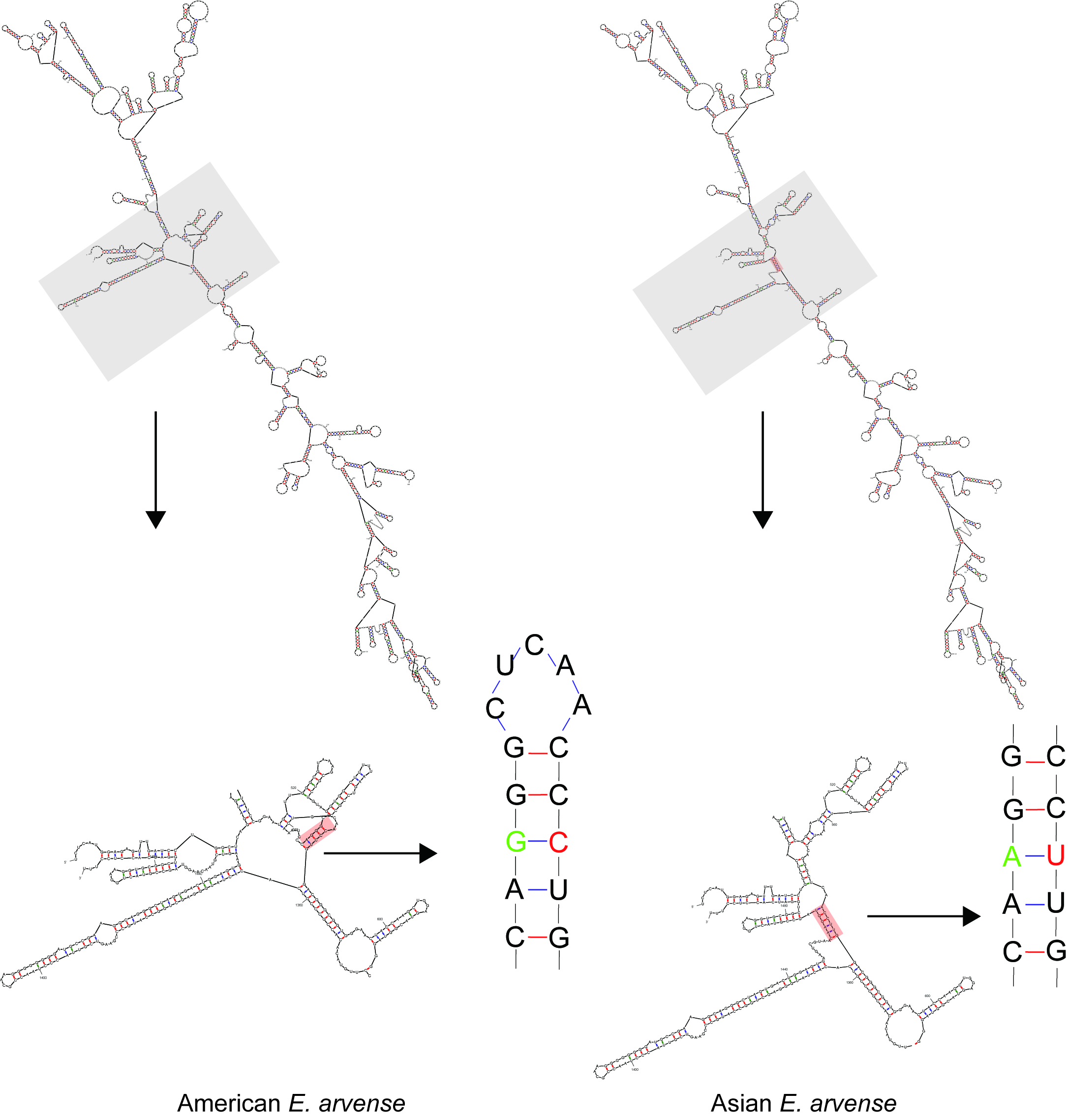

Supplement: Figure S2 — The RNA folding structure differences of rrn16 gene from two E. arvense populations. The gray box regions indicated the folding structure differences due to the SNP(C-U). The sequence -CCCUG- paired with the sequence –CAGGG- and form a hairpin structure in the American E. arvense (left). However, the sequence –CCUUG- paired with the sequence –CAAGG- and form a distinct stem structure in the Korean E. arvense (left). Two contrasting folding structures are based on the minimum free energy only. Other alternate folding structures are also possible if we consider other factors affecting the secondary structures. (TIF) [file pone.0103898.s002.tif]
